# Supplementary material for: Psychological Stress Reduces the Effectiveness of Periodontal Treatment: A Systematic Review
Source: J Clin Med. 2025 Mar 1;14(5):1680. doi: 10.3390/jcm14051680 (PMC11900564; doi:10.3390/jcm14051680)
Supplement: Supplementary file 1 [file jcm-14-01680-s001.zip › jcm-3469346-supplementary.pdf]

Table S1. Diagnosis —-periodontitis, psychological stress, biological biomarkers, inclusion and exclusion criteria, clinical parameters data (PPD, CAL, BOP, PI); main results, authors.

Authors and year of publication	***Methods of diagnosis***			***Inclusion/ exclusion criteria***	***Comparisons of clinical parameters according to the psychosocial context as well as the evolution after SRP***				***main results, authors'***
	Periodontal disease	***Psychological Stress (questionnaires/or biomarkers)***	***biological biomarkers***		***PPD (mean±SD)***	***CAL (mean±SD)***	***BOP (%) (mean±SD)***	***PI (%) (mean±SD)***	
Vettore et al., 2005 [31]	Chronic Periodontitis<br><br>-The control group had less than four sites with PPD ≤4.0 mm<br>-Test group 1 had at least four sites with PPD ≥4.0 and ≤6.0 mm<br>-Test group 2 had at least four sites with PPD >6mm	SSI	N.A	**IC**: age > 18 years; ≥ 14 teeth; minimum of 10 sites with PPD ≥ 5 mm.<br><br>**EC**: heavy Smoker (> 10 cigarettes/ day); systemic diseases (i.e., diabetes mellitus); intake of antibiotics, anti-inflammatory, or psychotropic; patients treated by NSPT in the last 6 months before recruitment, pregnant women and patients wearing orthodontic appliances.	Control Group<br>4mm to 6mm<br>**Mean of percentages**<br>Baseline<br>All subjects (n=20)<br>3.0±6.5<br>Stressed (n=6)<br>1.9±1.9<br>Unstressed (n=14)<br>3.5±6.2<br>3 months<br>All subjects (n=20)<br>3.2±6.7<br>Stressed (n=6)<br>4.8±1.9<br>Unstressed (n=14)<br>2.6±6.3	Control Group<br>4mm to 6mm<br>**Mean of percentages**<br>Baseline<br>All subjects (n=20)<br>9.0± 0.9<br>Stressed (n=6)<br>4.5±3.0<br>Unstressed (n=14)<br>10.8±10.2<br><br>3 months<br>All subjects (n=20)<br>9.0± 10.8<br>Stressed (n=6)<br>7.7±8.4<br>Unstressed (n=14)<br>9.7±11.9	Control Group<br>***Full mouth*** **percentages**<br>Baseline<br>All subjects (n=20)<br>6.4± 22.6<br>Stressed (n=6)<br>19.2±20.7<br>Unstressed (n=14)<br>15.3±24.1<br><br>3 months<br>All subjects (n=20)<br>11.0± 20.0<br>Stressed (n=6)<br>14.8±23.5<br>Unstressed (n=14)<br>9.5±19.3	Control Group<br>***Full mouth*** **Percentages** PI≥2<br>Baseline<br>All subjects (n=20)<br>56.5± 19.4<br>Stressed (n=6)<br>52.7±19.7<br>Unstressed (n=14)<br>58.3±19.8<br><br>3 months<br>All subjects (n=20)<br>11.4± 16.5*<br>Stressed (n=6)<br>9.8±9.1*<br>Unstressed (n=14)<br>12.2±19.2*	Periodontal measures were significantly different among the three groups at both baseline and 3 months after NPT analysis, except PI. The analysis of CAL and PPD periodontal sites categories of 4–6 and >6mm showed a gradual increase of deeper PPD and CAL frequencies, when the groups were compared. Frequencies of deeper PPD and CAL were higher in groups with more periodontal disease. BOP was also related to poorer periodontal condition.<br>A significant reduction in the frequencies of moderate (4–6 mm) and deep (>6 mm) PPD and CAL categories was observed 3 months after NSPT for group T2 (p<0.01). Similarly, moderate (4–6 mm) and deep (>6 mm)<br>PPD categories had reduced 3 months after NSPT for group T1. The percentage of sites

					Test Group 1<br>4mm to 6mm<br>percentages<br>Baseline<br>All subjects<br>(n=26)<br>20.6±11.7<br>Stressed (n=12)<br>24.6±11.2<br>Unstressed<br>(n=14)<br>17.3±11.6<br><br>3 months<br>All subjects<br>(n=26)<br>12.4±12.8*<br>Stressed<br>(n=12)<br>15.4±15.6*<br>Unstressed<br>(n=14)<br>9.8±9.9*<br><br>Test Group 2<br>> 6mm<br>percentages<br>Baseline<br>All subjects<br>(n=20)<br>42.0±17.6	Test Group 1<br>4mm to 6mm<br>percentages<br>Baseline<br>All subjects<br>(n=26)<br>27.4±15.2<br>Stressed<br>(n=12)<br>32.3±12.2<br>Unstressed<br>(n=14)<br>23.2±17.7<br><br>3 months<br>All subjects<br>(n=26)<br>22.0±14.2<br>Stressed<br>(n=12)<br>26.2±17.0<br>Unstressed<br>(n=14)<br>18.4±10.7<br><br>Test Group 2<br>PPD >6mm<br>percentages<br>Baseline<br>All subjects<br>(n=20)<br>47.3±15.2	Test Group 1<br>4mm to 6mm<br>percentages<br>Baseline<br>All subjects<br>(n=26)<br>39.3±29.0<br>Stressed<br>(n=12)<br>51.4±32.3<br>Unstressed<br>(n=14)<br>29.0±22.2<br><br>3 months<br>All subjects<br>(n=26)<br>23.0±27.0*<br>Stressed<br>(n=12)<br>34.1±34.1*<br>Unstressed<br>(n=14)<br>14.0±15.3*<br><br>Test Group 2<br>Baseline<br>All subjects<br>(n=20)<br>67.5±23.5<br>Stressed	Test Group 1<br>4mm to 6mm<br>percentages<br>Baseline<br>All subjects<br>(n=26)<br>51.0±21.0<br>Stressed<br>(n=12)<br>55.5±19.2<br>Unstressed<br>(n=14)<br>47.5±22.1<br><br>3 months<br>All subjects<br>(n=26)<br>20.3±20.9*<br>Stressed<br>(n=12)<br>19.6±20.3*<br>Unstressed<br>(n=14)<br>21.0±22.2*<br><br>Test Group 2<br>Baseline<br>All subjects<br>(n=20)<br>59.3±17.1<br>Stressed	with visible dental plaque and BOP dropped significantly in all groups.

					Stressed<br>(n=5)<br>46.2±22.8<br>Unstressed<br>(n=15)<br>40.4±16.3<br><br>3 months<br>All subjects<br>(n=20)<br>27.6±13.2*<br>Stressed<br>(n=5)<br>33.8±15.1<br>Unstressed<br>(n=15)<br>25.6±12.4*<br><br>Control Group<br>PPD >6mm<br><br>Baseline<br>All subjects<br>(n=20)<br>0.0±0.0<br>Stressed (n=6)<br>0.0±0.0<br>Unstressed<br>(n=14)<br>0.0±0.0<br>3 months<br>All subjects<br>(n=20)<br>0.1±0.3	Stressed<br>(n=5)<br>52.5±19.6<br>Unstressed<br>(n=15)<br>45.6±16.7<br><br>3 months<br>All subjects<br>(n=20)<br>39.0±19.0*<br>Stressed<br>(n=5)<br>49.2±19.1<br>Unstressed<br>(n=15)<br>35.7±18.6*<br><br>Control Group PPD >6mm<br><br>Baseline<br>All subjects (n=20)<br>0.5±1.0<br>Stressed (n=6)<br>0.3±0.7<br>Unstressed<br>(n=14)<br>0.5±1.2<br>3 months<br>All subjects (n=20)<br>0.7±2.0<br>Stressed (n=12)<br>0.2±0.4	(n=5)<br>69.9±31.8<br>Unstressed<br>(n=15)<br>67.1±21.5<br>3 months<br>All subjects<br>(n=20)<br>43.3±24.4*<br>Stressed<br>(n=5)<br>48.7± 32.4*<br>Unstressed<br>(n=15)<br>41.6±23.0*<br><br>Control Group<br>Mean difference<br>0-3 months<br>All subjects<br>5.4<br>stress level<br>4.4<br>Unstressed level<br>5.8<br><br>Group 1<br>Mean difference<br>0-3 months<br>All subjects<br>16.3<br>Stress level	(n=5)<br>58.1±18.3<br>Unstressed<br>(n=15)<br>59.8±17.4<br>3 months<br>All subjects<br>(n=20)<br>20.0±20.3*<br>Stressed<br>(n=5)<br>32.4± 28.5*<br>Unstressed<br>(n=15)<br>15.9±16.1*<br><br>Control Group<br>Mean difference<br>0-3 months<br>All subjects<br>45.1<br>stress level<br>42.9<br>Unstressed level<br>46.1<br><br>Group 1<br>Mean difference<br>0-3 months<br>All subjects<br>30.7<br>Stress level	

					Stressed (n=12)<br>0.3±0.4<br>Unstressed<br>(n=14)<br>0.1±0.2<br>Test Group 1<br>PPD >6mm<br>percentages<br>Baseline<br>All subjects<br>(n=26)<br>1.6±2.7<br>Stressed (n=12)<br>2.1±3.4<br>Unstressed<br>(n=14)<br>1.1±1.9<br>3 months<br>All subjects<br>(n=26)<br>0.6±1.3*<br>Stressed<br>(n=12)<br>0.9±1.6<br>Unstressed<br>(n=14)<br>0.4±1.2*<br><br>Test Group 2<br>PPD >6mm<br>Baseline	Unstressed<br>(n=14)<br>1.0±2.5<br>Test Group 1 PPD<br>>6mm<br>percentages<br>Baseline<br>All subjects<br>(n=26)<br>3.5±5.2<br>Stressed (n=12)<br>4.2±6.8<br>Unstressed<br>(n=14)<br>2.9±3.5<br>3 months<br>All subjects<br>(n=26)<br>2.7±4.5<br>Stressed<br>(n=12)<br>3.9±6.1<br>Unstressed<br>(n=14)<br>1.7±2.4<br>Test Group 2<br>CAL >6mm<br>Baseline<br>All subjects<br>(n=20)<br>20.7±15.4	17.3<br>Unstressed level<br>15<br>Group 2<br>Mean difference<br>0-3 months<br>All subjects<br>24.2<br>stress level<br>21.2<br><br>Unstressed level<br>25.5	35.9<br>Unstressed level<br>26.5<br>Group 2<br>Mean difference<br>0-3 months<br>All subjects<br>39.3<br>stress level<br>25.7<br><br>Unstressed level<br>43.9	

					All subjects (n=20)<br>14.4±10.2<br>Stressed (n=5)<br>11.2±3.4<br>Unstressed (n=15)<br>15.5±11.6<br><br>3 months<br>All subjects (n=20)<br>7.0±9.7*<br>Stressed (n=5)<br>3.5±4.1*<br>Unstressed<br>(n=15)<br>8.3±10.9*<br><br>Mean Difference<br>PD 4mm-6mm<br>Control Group<br>0-3 months<br>All subjects<br>-0.2<br>Stress level<br>+2.9<br>Unstressed level<br>-0.9<br><br>Group 1<br>Mean difference<br>0-3 months<br>All subjects<br>-8.2<br>Stress level<br>-9.2	Stressed(n=5)<br>16.6±5.9<br>Unstressed<br>(n=15)<br>22.1±17.5<br><br>3 months<br>All subjects<br>(n=20)<br>14.2±16.7*<br>Stressed<br>(n=5)<br>8.4±6.6<br>Unstressed<br>(n=15)<br>16.2±18.8*<br><br>Mean difference<br>PD 4mm-6mm<br>Control Group<br>0-3 months<br>All subjects<br>-0<br>stress level<br>-1.1<br>Unstressed level<br>-3.2<br><br>Group 1<br>Mean difference<br>0-3 months<br>All subjects<br>-5.4			

					Unstressed level<br>-7.5<br><br>Group 2<br>Mean difference<br>0-3 months<br>All subjects<br>-14.4<br>stress level<br>-12.4<br>Unstressed level<br>-14.8<br><br>Mean difference<br>>6mm<br>Control Group<br>0-3 months<br>All subjects<br>-0.0<br>stress level<br>-0.0<br>Unstressed level<br>-0.0<br><br>Group 1<br>Mean difference<br>0-3 months<br>All subjects<br>-1<br>Stress level<br>-1.2<br>Unstressed level<br>-0.7	stress level<br>-6.1<br>Unstressed level<br>-4.8<br><br>Group 2<br>Mean difference<br>0-3 months<br>All subjects<br>-8.3<br>stress level<br>-3.2<br>Unstressed level<br>-9.9<br><br>Mean difference<br>>6mm<br>Control Group<br>Mean difference<br>0-3 months<br>All subjects<br>+0.2<br>stress level<br>-0.1<br>Unstressed level<br>+0.5<br>Group 1<br>Mean difference<br>0-3 months<br>All subjects<br>-0.8<br>Stress level<br>-0.3			

					Group 2<br>Mean difference<br>0-3 months<br>All subjects<br>-7.4<br>stress level<br>-7.7<br>Unstressed level<br>-7.2	Unstressed level<br>-1.2<br>Group 2<br>Mean difference<br>0-3 months<br>All subjects<br>-2.4<br>stress level<br>-8.2<br>Unstressed level<br>-5.9			
Bakri et al., 2013	Chronic Periodontitis	PSS-10	Salivary cortisol<br><br>In GCF:<br>Elastase (ng/ml)<br>and ICTP (ng/ml)	**IC**: aged >20 years; no systemic disease that may affect periodontal disease progression or the outcome of treatment.<br>The smoking status (smoker or non-smoker) was recorded.<br>.	**Deep Bleeding sites**<br>Stressed group (n=29)<br>Baseline<br>6.8 ± 0.68<br>Unstressed group (n=16)<br>Baseline<br>6.8 ± 0.71<br>6 months<br>Stressed group (n=26)<br>-1.4 ± 0.93<br>Unstressed group (n=14)<br>-2.4 ± 0.76<br><br>**Deep non-Bleeding**<br>Stressed group (n=29)	**Deep Bleeding sites**<br>Stressed group (n=29)<br>Baseline<br>8.1 ± 1.15<br>Unstressed group (n=16)<br>Baseline<br>8.6 ± 0.99<br>6 months<br>Stressed group (n=26)<br>-1.1 ± 0.95<br>Unstressed group (n=14)<br>-2.3 ± 1.06<br><br>**Deep non- Bleeding**<br>Stressed group (n=29)<br>Baseline	N. A	N.A	Psychosocial stress is associated with a poorer outcome of NSPT based on clinical changes and biological markers.<br>Better results in terms of PPD and CAL changes at deep bleeding sites of patients with a low level of psychological stress using the PSS-10 scale. However, only two pocket sites were examined per patient, and plaque scores were not recorded.

					Baseline<br>6.3 ± 0.79<br>Unstressed group<br>(n=16)<br>Baseline<br>6.1 ± 0.46<br>6 months<br>Stressed group<br>(n=26)<br>-1.4 ± 1.08<br>Unstressed group<br>(n=14)<br>-1.8 ± 0.71	8.1 ± 1.34<br>Unstressed group<br>(n=16)<br>Baseline<br>7.5 ± 1.11<br>6 months<br>Stressed group<br>(n=26)<br>-1.7 ± 1.27<br>Unstressed group<br>(n=14)<br>-1.9 ± 1.09			
Varadhan et al., 2019 [33]	Chronic generalized periodontitis	DSP	Salivary cortisol	**IC**: age group of 30 to 55 years, systemically healthy individuals with chronic periodontitis.<br><br>**EC:** patients on corticosteroids and antipsychotic drug therapy, who had received any periodontal therapy, surgical, or NSPT within the past six months of baseline examination, smokers, no prior history of NSPT within 6 months, with less than	Stressed group<br>Baseline<br>(n=20)<br>6.98±1.33<br>Unstressed group<br>(n=20)<br>Baseline<br>6.31±1.92<br>3 months<br>Stressed group<br>(n=20)<br>6.69±1.54<br>Unstressed group<br>(n=20)<br>3.98±1.48*<br><br>Mean difference<br>0-3 months<br>Non stressed level	Stressed group<br>Baseline<br>(n=20)<br>3.76±1.75<br>Unstressed group<br>(n=20)<br>Baseline<br>3.17±1.73<br><br>3 months<br>Stressed group<br>(n=20)<br>3.61±1.79<br>Unstressed group<br>(n=20)<br>2.01±1.40*<br><br>Mean difference<br>0-3 months	BOP of PPD<br>Stressed group<br>Baseline<br>(n=20)<br>4.53±0.93<br>Unstressed group<br>(n=20)<br>Baseline<br>4.43±1.04<br>3 months<br>Stressed group<br>(n=20)<br>3.41±1.04<br>Unstressed group<br>(n=20)<br>1.69±0.76*<br><br>Mean difference<br>0-3 months	Stressed group<br>Baseline<br>(n=20)<br>1.12±0.16<br>Unstressed group<br>(n=20)<br>Baseline<br>1.22±0.35<br>3 months<br>Stressed group<br>(n=20)<br>1.05±0.13<br>Unstressed group<br>(n=20)<br>1.04±0.36*<br><br>Mean difference<br>0-3 months<br>Non stressed level	The reduction of total periodontitis-affected sites was highest in the group of non-stressed-level patients (55,4%), followed by stressed-level patients (38%).

				20 permanent teeth remaining, history of psychiatric treatment within past 6 months, known systemic diseases and conditions	2.32*<br><br>Stressed level<br>0.29	Non stressed level<br>1.16*<br><br>Stressed level<br>0.15	Non stressed level<br>2.74*[26]<br><br>Stressed level<br>1.12	0.18<br><br>Stressed level<br>0.07	
Ratika Lihala et al., 2019 [34]	Group I<br>Gingivitis<br><br>Group II<br>Chronic generalized periodontitis<br><br>Group III<br>Aggressive Generalized Periodontitis	GHQ	CgA levels	**IC:** age of 25–60 years, at least 20 natural teeth suffering from either mild gingivitis or PPD between 4–6mm and/or CAL of 3 -4mm<br>**EC:**<br>Patients with systemic diseases such as diabetes mellitus, rheumatoid arthritis, and renal disease, Pregnant and lactating, patients presenting with salivary gland dysfunction, undertaking antibiotics, steroids, or non-steroidal anti-inflammatory medication within the past three months, Smokers, Drugs which	PPD<br>Group I<br>N.A<br><br>Group II<br>Stress Level (GHQ: 14.8)<br>Baseline<br>6.87±0.41<br>Group III<br>Stress level (GHQ:16.8)<br>6.9±0.54<br>3 months<br>Group II<br>Stress level (GHQ:14.27)<br>5.13±0.43*<br>Group III<br>Stress level (GHQ:13.8)<br>5.57±0.48*<br><br>Mean difference<br>Grupo II	Group I<br>N.A<br><br>Group II<br>Stress Level (GHQ: 14.8)<br>Baseline<br>6.11±0.70<br>Group III<br>Stress level (GHQ:16.8)<br>6.41±0.49<br>3 months<br>Group II<br>Stress level (GHQ:14.27)<br>4.06±0.32*<br>Group III<br>Stress level (GHQ:13.8)<br>4.99±0.34*<br><br>Mean difference<br>Grupo II	N.A	Group I<br>Stress Level (GHQ: 8.2)<br>Baseline<br>0.41±0.06<br>Group II<br>Stress Level (GHQ: 14.8)<br>Baseline<br>2.17±0.23<br>Group III<br>Stress level (GHQ:16.8)<br>1.21±0.43<br>3 months<br>Group I<br>Stress Level (GHQ: 7.50)<br>0.29±0.04*<br>Group II<br>Stress level (GHQ:14.27)<br>0.79±0.16*<br>Group III<br>Stress level	Based on the post hoc test results, the GHQ score was the highest in CP and AgP groups compared to the gingivitis group at baseline and three months, post-treatment.<br>Post-NSPT improvement in the GHQ scores was statistically highly significant for both the CP (P <0.006) and AgP (P <0.001) groups.<br>A statistically significant correlation was established between levels of CgA and stress parameters, which was shown to be the highest in AgP (P<0.001), followed by the CP group (P< 0.005) at baseline. Amongst the

				interfere with salivary secretions	1.74<br>Grupo III<br>1.33	2.05<br>Grupo III<br>1.42		(GHQ:13.80)<br>0.43±0.18*<br><br>Mean difference<br>Grupo I<br>0.12<br>Grupo II<br>2.05<br>Grupo III<br>1.42	clinical parameters, CAL showed the strongest correlation with CgA both at baseline and after NSPT (P< 0.001).<br>NSPT showed a marked improvement in all the parameters. Levels of CgA and CAL showed a significant correlation in both the CP and AgP groups.
Dubar et al., 2020 [32]	Periodontitis stage III/IV grade A-C<br><br>Healthy control (without periodontitis)	PSS and STAI-Y	salivary cortisol	**IC:** aged ≥ 18; moderate to severe chronic periodontitis; with at least two PD ≥ 5 mm.<br><br>**EC:** without pathology; no history of taking antimicrobials, or anti-inflammatory drugs; not pregnant; no NSPT in the previous 6 months.<br>-Control group: absence of periodontal disease; in good health; not pregnant.	Test Group Periodontal patients Median (Min-Max)<br>Highly stressed (N=14)<br>Baseline<br>6.0[1.0–11.0]<br>Mean ± SD<br>6.0±2.92<br><br>Non-Highly stressed (N=16)<br>6.5[1.0–10.0]<br>Mean ± SD<br>6.1±2.63	Test Group<br>Periodontal patients<br>Median (Min-Max)<br>Highly stressed (N=14)<br>Baseline<br>6.0[1.0–11.0]<br>Mean ± SD<br>6 ± 2.92<br><br>Non-Highly stressed<br>7.0[1.0–16.0]<br>Mean ± SD<br>7.5 ±4.39<br><br>Control Group	Test group<br>All patients<br>Before SRP<br>60<br>After SRP<br>48.3<br><br>Control Group<br>All patients with and without stress<br>Before SRP<br>0.0<br>After SRP<br>0.0	Test Group Periodontal patients<br>PI scores%<br>Highly stressed<br>Baseline<br>1= 19<br>2= 26.2<br>3= 4.8<br>Non-Highly stressed<br>1= 37.5<br>2= 14.6<br>3= 2.1<br><br>Psychosocial context of control patients	Before SRP, significant differences between pathological and healthy sites or control sites were observed for each parameter (Pl, GI, BOP, PPD, CAL). After SRP, comparisons of periodontal parameters still indicate significant difference between healthy and pathological sites from periodontitis patients about all parameters (p < 0.001).

					Control Group<br><br>Highly stressed (N=14)<br>Baseline Median (Min-Max)<br>1.5[1.0–2.0]<br>Mean ± SD<br>1.5 ± 0.29<br><br>Non-Highly stressed<br>(n=16)<br>1.0[1.0–3.0]<br>Mean ± SD<br>1..3± 0.56<br>Test Group<br>All patients<br>Before SRP<br>7[5–11]<br>Mean ± SD<br>7.23±1.47<br><br>After SRP<br>(15 weeks)<br>5[2–13]<br>Mean ± SD<br>5.5±2.70<br><br>Control Group	Highly stressed (N=14)<br>Baseline % Median (Min-Max)<br>1.0[1.0–2.0]<br>Mean ± SD<br>1.17 ± 0.29<br><br>Non-Highly (n=16)<br>stressed<br>2.0[1.0–3.0]<br>Mean ± SD<br>2 ± 0.57<br>Test Group<br>All patients<br>Before SRP<br>Baseline<br>8[5–16]<br>Mean ± SD<br>8.59 ± 2.69<br><br>After SRP<br>6[3-20]<br>Mean ± SD<br>7.3±4.16<br><br>Control Group<br>All patients<br>with and without stress<br>Before SRP<br>2[1–5]		Highly stressed (N=14)<br>Baseline % Median (Min-Max)<br>0: 92.9<br>1: 7.1<br>Non-Highly<br>(n=16)<br>stressed<br>0: 87.5%<br>1: 12.5%<br><br>Control Group<br>All patients<br>with and without stress<br>Before SRP<br>0.0<br><br>After SRP<br>0.0<br><br>Test Group<br>All patients<br>Before SRP<br>5.0<br><br>After SRP<br>3.3	

					All patients with and without stress<br>Before SRP<br>2[1–3]<br>Mean ± SD<br>(15 weeks)<br>2±0.49<br>After SRP<br>2[1–3]<br>Mean ± SD<br>2±0.49	Mean ± SD<br>2.24 ± 0.98<br><br>After SRP<br>2[1–6]<br>Mean ± SD<br>2.35 ± 1.23			
Bebars et al., 2021 [29]	Periodontitis stage III/IV grade A/B	PSS-10 and SVS	N. A	**IC:** age > 18 years; presence of ≥ 14 teeth; a minimum of 10 sites with PPD ≥ 5 mm.<br><br>**EC:** heavy smoker (> 10 cigarettes/ day); systemic diseases (i.e., diabetes mellitus); intake of antibiotics, anti-inflammatory, or psychotropic medications; patients treated by NSPT in the last 6 months before recruitment, pregnant women and patients with orthodontic appliances.	Low-stress level (n=22)<br>Baseline<br>4.0±0.8<br><br>High stress level (n=33)<br>3.9±0.7<br><br>6 weeks<br><br>Low-stress level (n=22)<br>3.1±0.6<br><br>High-stress level (n=33)<br>3.0±0.4<br><br>Mean difference<br>0-6 Weeks<br>Low-stress level<br>0.9<br><br>High-Stress Level<br>0.9	Low- stress level (n=22)<br>Baseline<br>4.8±1.3<br><br>High-stress level (n=33)<br>4.7±0.9<br>6 weeks<br><br>Low-stress level (n=22)<br>4.1±1.4<br><br>High- stress level (n=33)<br>4.1±0.7<br><br>Mean difference<br>0-6 Weeks	*Full mouth*<br>Low stress level (n=22)<br>Baseline<br>64.9±24.6<br>High stress level (n=33)<br>61.1±21.5<br>6 weeks<br><br>Low-stress level (n=22)<br>33.2±12.7<br><br>High-stress level (n=33)<br>20.4±14.5*<br><br>Mean difference<br>0-6 Weeks<br>Low-stress level	*Full mouth*<br>Low stress level (n=22)<br>Baseline<br>68.1±20.2<br>High stress level (n=33)<br>62.8±20.1<br>6 weeks<br><br>Low-stress level (n=22)<br>22.0±12.1<br><br>High-stress level (n=33)<br>26.8±13.9<br><br>Mean difference<br>0-6 Weeks<br>Low-stress level<br>46.1	At 6-week follow-up, both groups showed an improvement in the level of their oral hygiene with comparable mean FMPS values (26.8 ± 13.9% in group 1 versus 22.0 + 12.1% in group 2). The influence of stress on the individual inflammatory response was highlighted. FMBS underwent a larger contraction in groups of low stress compared to groups of high stress, reaching an average percentage of 20.4 + 14.5%, respectively, versus 33.2 + 12.7%. Regarding the decrease in PPD and increase in CAL, there were no statistically significant differences between the two groups

						Low - stress level<br>0.7<br><br>High-Stress Level<br>0.6	31.7<br><br>High-Stress Level<br>40.7	High-Stress Level<br>36	
Petit et al., 2021 [19]	Severe chronic periodontitis stage III/IV generalized	DASS-42 and TCS	Plasma levels of cortisol(ng/ml) and CgA(ng/ml)	**IC**: age > 18 years; > 15 teeth; 5% of sites with PD > 5 mm and radiographic bone loss.<br><br>**EC:** systemic diseases; history of taking anti-inflammatory drugs, psychotropic drugs, and antibiotics in the past 6 months; pregnancy; orthodontic therapy; history of NSPT in the past 6 months	Baseline (n=71)<br>3.82±0.68<br><br>3months (n=58)<br>3.23±0.59<br><br>6 months (n=54)<br>3.09±0.57<br><br>T0-T3<br>-0.59±0.09<br><br>T0-T6<br>-0.73±0.11<br><br>ΔPPD<br>0.01 (0.005)	Baseline<br>(n=71)<br>5.07±1.15<br><br>3months<br>(n= 58)<br>4.47±1.13<br><br>6 Months<br>(n= 54)<br>4.44±1.13<br><br>T0-T3<br>-0.60±0.02<br><br>T0-T6<br>-0.63±0.02<br><br>ΔCAL<br>0.01 (0.008)	*Full mouth*<br>Baseline<br>(n=71)<br>0.63±0.21<br><br>3months<br>(n=58)<br>0.36±0.19<br><br>6 months<br>(n=54)<br>0.28±0.18<br><br>T0-T3<br>-0.27±0.02<br><br>T0-T6<br>-0.35±0.03<br>ΔBOP<br>1.02 (1.00-1.04)	*Full mouth*<br>Baseline<br>(n=71)<br>1.3±0.51<br><br>3months<br>(n=58)<br>0.75±0.51<br>6 months<br>(n=54)<br>0.61±0.40<br><br>T0-T3<br>-0.55±0.0<br><br>T0-T6<br>-0.69±0.11<br>ΔPI<br>0.01 (0.00)	An average PPD reduction of 0.73+/-0.11mm was measured at 6 months. The same trend of results was observed when considering CAL, BOP, and PI (*p*<0.05).
Romano et al., 2023 [18]	Generalized stage III/IV grade A/B periodontitis	PSS-10 and CRI-Adult	N. A	**IC:** age > 18 years; presence of ≥ 14 teeth; a minimum of 10 sites with PPD ≥ 5 mm.<br>**EC:** heavy smoker (> 10 cigarettes/ day); systemic diseases (i.e.,	Baseline<br>Minor stress level<br>(n=63)<br>Baseline<br>3.8±0.9<br>Major stress level<br>(n=27)<br>4.0±0.6<br>3 months	Baseline<br>Minor stress level<br>(n=63)<br>Baseline<br>4.4± 1.4<br><br>Major stress level<br>(n=27)<br>4.7± 1.3	*Full mouth*<br>Baseline<br>Minor stress level<br>(n=63)<br>Baseline<br>(FMBS)<br>66.1± 23.9<br><br>Major stress level	*Full mouth*<br>Baseline<br>Minor stress level<br>(n=63)<br>Baseline<br>(FMPS)<br>68.7± 20.3<br><br>Major stress level	All clinical parameters were similar at the baseline across different categories. At re-evaluation, reduction means PPD and CAL were higher in the minor stress group (p =0.001 and p =0.013, respectively), as well

				diabetes mellitus); intake of antibiotics, anti-inflammatory, or psychotropic medications; patients treated by NSPT in the last 6 months before recruitment, pregnant women and patients with orthodontic appliances.	Minor stress level (n=63)<br>2.9±0.5<br>Major stress level (n=27)<br>3.3±0.5<br>Sites with PPD ≥6mm<br>Baseline<br>Minor stress level (n=63)<br>26.2±20.8<br>Major stress level (n=27)<br>29.8±24.6<br><br>3 months<br>Minor stress level (n=63)<br>8.1± 6.7<br>Major stress level (n=27)<br>12.5±8.8<br><br>Sites with PPD 4-5 mm<br>Baseline<br>Minor stress level (n=63)<br>48.8±21.6<br>Major stress level (n=27)<br>28.3±18.1<br>3 months<br>Minor stress level (n=63)<br>27.4±16.2<br>Major stress level (n=27)<br>32.3±15.6<br><br>Sites with PPD ≤3mm<br>baseline<br>Minor stress level (n=63)<br>82.5±29.9<br>Major stress level (n=27)<br>77.0±28.6	3 months<br>Minor stress level (n=63)<br>3.8± 1.0<br>Major stress level (n=27)<br>4.4 ± 1.2<br><br>Mean difference<br>0-3 months<br>Minor stress level<br>0.6<br><br>Major Stress level<br>0.3	(n=27)<br>69.6±21.6<br>3 months<br>Minor stress level (n=63)<br>20.2± 8.2<br>Major stress level (n=27)<br>39.8 ± 12.8<br><br>Mean difference<br>0-3 months<br>Minor stress level<br>45.9<br><br>Major Stress level<br>29.8	(n=27)<br>69.5±22<br>3 months<br>Minor stress level (n=63)<br>20.2± 10.2<br>Major stress level (n=27)<br>24.1 ± 10.9<br><br>Mean difference<br>0-3 months<br><br>Minor stress level<br>48.5<br><br>Major Stress level<br>45.4	as mean number of PPD ≥ 6 mm (p =0.020).<br>FMPS did not differ between the 2 groups, whereas FMBS resulted significantly more in the minor stress group (p <0.001).<br>Conversely, the mean number of teeth did not show a significant differential change between the groups.

					3 months<br>Minor stress level (n=63)<br>119.2±29.6<br>Major stress level (n=27)<br>106.8±23.5<br><br>Mean difference<br>0-3 months<br>Minor stress level<br>0.9<br><br>Major Stress level<br>0.7<br>Mean difference<br>PPD > 6mm<br>0-3 months<br>Minor stress level<br>18.1<br>Major Stress level<br>17.3<br>Mean difference<br>PPD 4- 5mm<br>0-3 months<br>Minor stress level<br>21.4<br>Major Stress level<br>-4<br>Mean difference<br>PPD <3mm<br>0-3 months<br>Minor stress level				

					-37<br>Major Stress level<br>-29				

PPD, probing pocket deep; CAL, clinical attachment level; BOP, bleeding on probing; PI, plaque index; GCF, gingival crevicular fluid; PSS, Perceived Stress Scale questionnaire; SSI, Stress Symptoms Inventory; SRRS, Social Readjustment Rating Scale; STAI-Y, State Anxiety Inventory; SVS, Stress-Related Vulnerability Scale; DSP, Derogatis Stress Profile; GHQ, The General Health Questionnaire; DASS-42, Depression Anxiety Stress Scale 42; TCS, Toulouse Coping Scale; CRI-Adult, Coping Responses Inventory-Adult form; ICTP, C-terminal teleopeptide of type collagen; CgA, chromogranin A; IC, Inclusion criteria; EC, exclusion criteria; SRP, scaling root planing; NSPT, Non-Surgical Periodontal Therapy; N.A, no evaluated.
